# A System-Oriented Dialogue Model to Design Community Partnerships for More Effective Sars-Cov-2 Prevention in Schools: The Case of Spain

**DOI:** 10.3389/ijph.2023.1605624

**Published:** 2023-05-02

**Authors:** Rosina Malagrida, Jessica Fernández, Jordi Casabona, Jacqueline E. W. Broerse

**Affiliations:** ^1^ Living Lab for Health, IrsiCaixa AIDS Research Institute, IGTP, Badalona, Spain; ^2^ Athena Institute, Vrije Universiteit Amsterdam, Amsterdam, Netherlands; ^3^ Centre d’Estudis Epidemiològics sobre les Infeccions de Transmissió Sexual i Sida de Catalunya (CEEISCAT), Barcelona, Spain; ^4^ Centro de Investigación Biomédica en Red de Epidemiología y Salud Pública (CIBERESP), Barcelona, Spain

**Keywords:** COVID-19 prevention, system analysis, community-based participatory research (CBPR), integrated intervention, community partnership, engagement

## Abstract

**Objectives:** The European Centre for Disease Control (ECDC) COVID-19 guidelines for non-pharmaceutical interventions (NPI) identify safety, hygiene and physical distancing measures to control SARS-Cov-2 transmission in schools. Because their implementation requires complicated changes, the guidelines also include “accompanying measures” of risk communication, health literacy and community engagement. Although these are considered crucial, their implementation is complex. This study aimed to co-define a community partnership that a) identifies systemic barriers and b) designs recommendations on how to implement the NPI to improve SARS-Cov-2 prevention in schools.

**Methods:** We designed and piloted a System-Oriented Dialogue Model with the participation of 44 teachers and 868 students and their parents from six Spanish schools during 2021. The results were analysed using thematic analysis.

**Results:** Participants identified 406 items addressing issues related to system characteristics, which is indicative of the complexity of the challenge. Using a thematic analysis, we defined 14 recommendations covering five categories.

**Conclusion:** These findings could help in developing guidelines for initiating community engagement partnerships in schools to provide more integrated prevention interventions.

## Introduction

The European Centre for Disease Prevention and Control (ECDC) stated in 2020 that closing schools to control the COVID-19 pandemic should be done as a last resort due to its negative impact on children’s physical and mental health and education as well as its broader economic impact on society. It therefore developed guidelines for non-pharmaceutical interventions (NPI) aimed at preventing and controlling SARS-Cov-2 transmission in school settings (1, 2). These include safety- and hygiene-related measures—such as using masks, appropriate ventilation and respiratory and hand hygiene; ensuring appropriate facility cleaning and promoting a “stay at home when sick” policy—and physical distancing measures—such as creating cohorts of classes and groups; ensuring physical distancing in the classroom; reducing class sizes; staggering arrival, meal and break times or holding classes outdoors. Schools are advised to adapt the physical distancing measures to the setting and age group and to balance preventing transmission with providing optimal learning and psychosocial environments. The ECDC also recommends that such measures be accompanied by risk communication, health literacy and community engagement, including the voices of the children and other stakeholders. These “accompanying measures” are considered crucial components of an effective response in school settings (1, 2). They are also being promoted by the WHO and UNESCO within an initiative called Health Promoting Schools (3, 4).

Although most of these measures have been implemented with a high degree of effectiveness that has allowed many EU countries to keep schools open for long periods during the pandemic (5), their practical implementation has been complex (6). Challenges include how to find the right balance between preventing transmission and providing children with optimal environments, how to implement the accompanying measures or how to respond to more practical issues such as coping with physical distancing in spaces that do not allow it (1, 2).

These challenges are also complex, and they will require systemic changes in schools (7), such as changes in a) current routines and ways of working, b) rules, policies, guidelines, power structures and distribution of resources and c) mental and cultural models used within a school setting, including norms, values, beliefs, attitudes and expectations. Such changes are often initially overlooked and may subsequently give rise to systemic barriers. This is because systems actively create barriers for interventions that are incompatible with the dominant cultures, structures and practices within them (7). If these systemic barriers are not adequately addressed, the prevention measures cannot be implemented effectively. The ECDC, WHO and UNESCO have therefore suggested a new model for health protection and promotion for use within community partnerships in which different social actors from the education community work together to design and implement effective solutions for the emerging challenges (1–4). However, there are no clear guidelines on how to set up participatory community partnerships while assuring they will lead to the identification and implementation of appropriate (accompanying) measures that consider the systemic barriers. Our research therefore aimed to design and pilot a System-Oriented Dialogue Model for a) identifying the systemic barriers to implementing NPI for SARS-Cov-2 prevention in schools and b) co-designing recommendations on how to address those barriers within community partnerships.

## Methods

This research was conducted within the framework of the broader Sentinel Schools Study, which forms part of the Catalonia Ministry of Health’s COVID-19 prevention plan. The broader study aims to monitor and evaluate the COVID-19 pandemic in school settings and to identify barriers and facilitators for improving prevention measures. It is run by a consortium of experts in healthcare and in public health research who were consulted during different phases of the process.

We used a Community-Based Participatory Research (CBPR) approach to co-design the recommendations for setting up community partnerships because both share the same principles: the participation and empowerment of different community members and the relevance and impact of research. In CBPR, research participants from a community are actively involved in the research process and the research is action oriented, which is especially useful when the goal is to achieve real-life change (8). For this project, we also used the Dialogue Model (9), as it describes a structured multistakeholder process for designing action agendas to address a problem. In addition, its principles are also aligned with those of CBPR: active engagement of stakeholders, respect for experiential knowledge and dialogue between stakeholders. The model provides clear prescriptive guidelines on how to consult with various stakeholders and integrate their perspectives, and it has been validated by numerous case studies on a wide range of health issues (10). It comprises the following phases: Exploration, Consultation, Prioritization, Integration, Programming and Implementation. We adapted the model in two ways. First, we swapped the order of the prioritization and integration phases so the priority-setting process would be based on all the results, although this phase and the programming and implementation phases were beyond the scope of our project. We also added a new phase—Dissemination—to follow integration because we considered it essential to validate and discuss the results with participants and then disseminate them to key stakeholders who could contribute to the last three phases of the model. Second, as the Dialogue Model does not explicitly use a systems approach, we included a systems analysis to ensure we could identify the systemic barriers. For this analysis, we applied Van Mierlo et al.’s (11) system analysis tool, which classifies the barriers to system transformation according to the different characteristics of a system. To fit our topic and actors, we added a new characteristic named “health,” renamed “market structure” as “economy” and added information to contextualize each of the characteristics (see Box 1).

BOX 1 **System characteristics to be analysed during the consultation phase (adapted from Van Mierlo et al’s, 2010, system analysis tool). System-Oriented Dialogue Model, Catalonia, Spain, 2021.**

**“Access to knowledge/information and research development”**: education, communication, access to contrasted information, etc.

**“Physical and virtual infrastructure”**: school, family and leisure spaces, new technologies, etc.

**“Legislation, regulation and compliance”**: from the government and from the school and social environment, such as handwashing, ventilation, social distancing, etc.

**“Roles and interaction and collaboration between different actors”**: between teachers, students, families, administration, health sector, social environment, etc.

**“Economy”**: changes in families’ employment situations, resources to deal with the changes, etc.

**“Health”**: impact on physical and psycho-social health, agility of response with preventive measures, perception of risk by different social actors, willingness to be vaccinated, etc.

**“Values, norms and symbols”**: refers to a country’s, region’s or sector’s culture, its social norms and values and its political and economic climates. This characteristic is key because it generates problems and needs within the former system characteristics, which mostly refer to structures, patterns and events (12).

We called this novel methodology the System-Oriented Dialogue Model.

**FIGURE 1:**
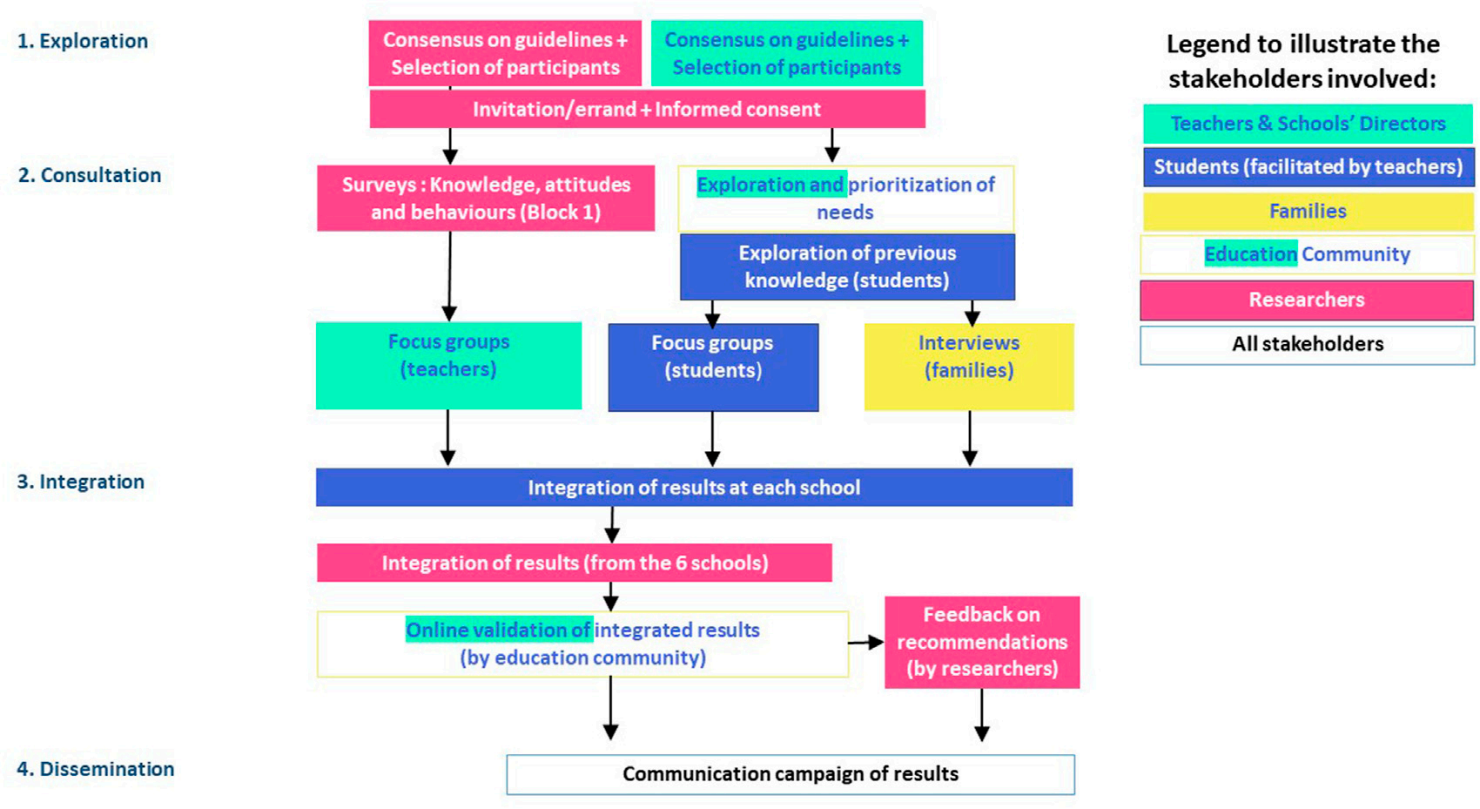
System-Oriented Dialogue Model workflow. System-Oriented Dialogue Model, Catalonia, Spain, 2021.

The System-Oriented Dialogue Model was implemented from January to June 2021 with six participating schools from the Sentinel School Network in Catalonia, Spain (including both primary and secondary schools) (see Figure 1). The schools were identified within a school sentinel surveillance network created within the Sentinel Schools Study and were selected considering socio-economic, rural-urban and private-public criteria to ensure a diverse sample. Below, we describe the activities in the different phases.

### Phase 1: Exploration

In the exploration phase, we operationalized the System-Oriented Dialogue Model by defining guidelines and concrete activities for the different phases to be implemented with teachers, students (aged 10–17 years) and their parents. The guidelines described a) how researchers could engage teachers, students and parents, b) how researchers could conduct focus group discussions (FGDs) with teachers, c) how teachers could conduct FGDs with students, and d) how students could interview their parents. These guidelines also included complementary educational activities to stimulate students’ learning and optimal participation. All guidelines were designed to ensure that the group dynamics promoted collective deliberation.

Once the guidelines were validated by other researchers from the Sentinel Schools Study, we presented the project to schools *via* online meetings.These were followed by mailings to directors and teachers where they were invited to suggest changes. Their suggestions were used improve the final guidelines. These stakeholders were prioritized during this phase to ensure that the final design fitted their needs and expectations so as to facilitate their involvement in the implementation.

### Phase 2: Consultation

This phase started with six online FGDs with teachers, one in each school. A total of 44 teachers from 7 primary and secondary schools participated. Each FGD began with a presentation of the project, its aims and methodology, followed by a presentation on the topic of COVID-19 prevention in schools. The teachers were then invited to follow the steps of the model’s guidelines. The FGDs lasted approximately 120 min.

After the FGDs, the teachers received the guidelines for organizing a consultation process with students and their parents. Of the 44 teachers who attended the online FGDs, 22 conducted FGDs with their students. Those who did not were teachers and school heads with no students in the designated age range for participation, and teachers that were not able to complete the work within the deadlines defined in the guidelines. The participating teachers used communication materials to introduce the project and invite their students to participate. These included a letter of invitation/errand in which researchers from the Sentinel Schools Study asked the students and their parents to participate as co-researchers together with the teachers.

The guidelines for students began with preparatory educational activities inviting students and their parents to familiarize themselves with the subject (e.g., by reading articles on the topic related with the different characteristics of the system analysis). After these preparatory tasks, the teachers conducted FGDs with their students. A total of 868 students participated: 122 in primary education, 648 in lower secondary education and 98 in upper secondary education (Table 1). Subsequently, the students interviewed their parents.

**TABLE 1:** Distribution of participating students across education levels and of teachers participating in online Focus Groups and facilitating Focus Groups with students. System-Oriented Dialogue Model, Catalonia, Spain, 2021.

Schools	Teachers participating in online focus groups	Teachers facilitating focus groups with students	Primary students	Lower secondary students	Upper secondary students
School 1	7	3	51	0	0
School 2	10	4	71	0	0
School 3	8	0	0	0	0
School 4	8	8	0	260	0
School 5	6	3	0	118	0
School 6	5	4	0	270	98
Total	44	22	122	648	98

### Phase 3: Integration

Class specific FGDs and interview results were integrated during a session with each school class. In these teacher-facilitated sessions, the results from the students’ FGDs were integrated with the results from the parents’ interviews and those from the online FGD facilitated by researchers.

The integrated results from each school class were sent to the first two authors of this paper. They checked the submitted tables for completeness by comparing them with the results from the FGDs with teachers; any missing topics were added to the tables. The results from all classes were then integrated into one final preliminary list of problems, needs and recommendations. These were classified into five main categories and then further divided into clusters using a thematic analysis. The preliminary integrated and structured list of recommendations was sent back to the participants for final validation through an online questionnaire. In total, 280 responses were received from 21 teachers, 236 students and 23 parents. These resulted in some minor modifications before a final list of recommendations was obtained. Researchers from the Sentinel Schools consortium also shared feedback on the final recommendations; their comments were added as footnotes to the recommendation list.

We also conducted a frequency count to determine if the participatory system analysis had been effective at identifying key problems and needs in the system characteristics within each category, as these could act as systemic barriers. The frequency analysis is important because the number of characteristics mentioned per category and the weight of each is indicative of each category’s complexity. When there is high complexity, one-off interventions that address problems or needs in isolation (i.e., in one part of a category) are usually not effective enough (12). For this analysis, we calculated the percentage of items per cluster and assigned that percentage to each system characteristic covered by that cluster. Thus, the coverage of each characteristic was weighted against the percentage of items in that cluster compared to the other clusters in the same category. This allowed us to visualize the “weight” that each system characteristic had within each cluster and category.

Finally, a second thematic analysis was conducted and the results were classified into two categories—thematic and process-oriented recommendations—to facilitate moving towards the phases of prioritization, programming and implementation.

### Phase 4: Dissemination

Students presented the edited list of recommendations to scientists and policymakers in June 2021 during an online congress attended by 241 students, 16 teachers, 15 researchers and healthcare providers and 3 policymakers. During the congress, the experts shared their views, based on scientific evidence, and added comments to the final list of recommendations, as described in Phase 3. The results were also disseminated to policymakers and to the education community through other channels.

As no personal data was collected for the study, no ethics approval or specific consent procedures were required.

## Results

The items identified with the System-Oriented Dialogue Model were distributed into categories and clusters of problems, needs and recommendations using the thematic analysis. In this section, we first describe those problems and needs within each category that act as systemic barriers to the implementation of prevention measures and should therefore be addressed through accompanying measures. Then we present the analysis of how the items were distributed across the system characteristics. We finish by describing the clusters of priority recommendations within each category that should be addressed as accompanying recommendations. All the problem and needs, clusters, recommendations and recommendation clusters are presented in Supplementary File S1.

### Identified Problems and Needs

A total of 406 items describing the problems and needs were collected and distributed into 40 clusters and 5 categories using thematic analysis (see Table 2). Brief descriptions of the categories are provided here.

1. *Participation of the education community and other stakeholders*: comprises problems related to transitioning towards a more open and inclusive model of governance of the pandemic and of research and innovation (R&I). The cluster with the most items (37) corresponds to the “Lack of participation in decision-making at political level and within schools and other environments to adapt regulations to each context, to promote co-responsibility and to reach consensus on the needs for R&I.”
2. *Physical, mental and social health*: includes problems and needs related to mental and social health (e.g., social relationships, loneliness) and to physical health (e.g., the pandemic’s impact on physical exercise, weight gain and obesity, skin condition, breathing and vocal cords). The main cluster (with 29 items) corresponds to the “Impact of COVID-19 on mental health,” followed by clusters on the “Inconveniences of using masks on communication, hygiene, breathing, pressure on ears and vocal cords, etc., for the different stakeholders” (18 items) and the “Impact of COVID-19 on social health of students outside school” (16 items).
3. *Infrastructures and waste management*: includes problems and needs in implementing ventilation and social distancing measures and problems involving the waste generated by control measures (e.g., masks and self-tests). The main cluster (12 items) corresponds to “Difficulties with effective implementation of ventilation measures”: for example, how to adapt those measures when it is noisy or cold outside or when the number of students per class is higher than recommended.
4. *Communication and education for prevention*: comprises problems and needs related to fragmented communication and education campaigns and to education models. It includes the need to better address pandemic-related competencies within formal and non-formal education, such as the ability to discern between evidence-based and non-evidence-based information or to increase citizenship responsibility. The main cluster (32 items) corresponds to “Ineffective communication: confusing, too focused on risks, contributing to stigma of certain age groups, non-transparent, non-evidence-based, overloading, inaccessible to certain groups.” This was followed by “Online classes are least effective and cause more fatigue” (31 items), which focused mostly on problems concerning the model of education, and the “Lack of awareness of the pandemic’s consequences to realize the importance of complying with regulations” (29 items).
5. *Social inequalities*: comprises problems and needs related to social and digital inequalities. The main cluster (35 items) corresponds to the “Impact of COVID-19 on social inequalities,” followed by the “Impact of digital divide during COVID-19” (29 items).

**TABLE 2:** Problems and needs categories and clusters, including the number and percentage of items per cluster and the weight of individual system characteristics within each cluster and category. System-Oriented Dialogue Model, Catalonia, Spain, 2021.

Categories	Clusters of problems and needs	Number of items per cluster	Percentage of items per cluster	System characteristics (weighted average by percentage)						
				Access to knowledge, information and research development	Physical and virtual infrastructure	Legislation and regulation	New roles and interactions and collaborations between actors	Economy	Health	Values, norms and symbols
1: prevention with participation of the education community and other stakeholders	Lack of participation in decision-making at political level and within schools and other environments to adapt regulations to each context, to promote co-responsibility and to reach consensus on the needs for R&I	37	9.1	9.1	0	9.1	9.1	0	0	9.1
	Norms are not equitable between education levels^a^	2	0.5	0	0	0.5	0	0	0	0.5
	Norms are not adapted to the COVID-19 prevalence in different geographic zones	4	1.0	0	0	1.0	0	0	0	1.0
	Failure to comply with prevention measures	2	0.5	0	0	0.5	0	0	0	0
Total category 1	**4**	**45**	**11.1**	**9.1**	**0**	**11.1**	**9.1**	**0**	**0**	**10.6**
2: Physical, mental and social health	It takes too long to receive PCR test results	7	1.7	0	1.7	1.7	0	1.7	1.7	0
	Impact of COVID-19 on physical exercise	4	1.0	0	0	0	0	0	1.0	0
	Impact of COVID-19 on weight gain and obesity	5	1.2	0	0	0	0	0	1.2	0
	Inconveniences of using masks (on communication, hygiene, breathing, pressure on ears and vocal cords, etc.) for the different stakeholders	18	4.4	0	0	0	0	0	4.4	0
	Impact of hydroalcoholic gels on skin	3	0.7	0	0	0	0	0	0.7	0
	Students need to transport a lot of books due to COVID-19 measures	1	0.2	0	0	0	0	0	0.2	0
	Impact of COVID-19 on screen addictions	6	1.5	0	0	0	0	0	1.5	0
	Impact of COVID-19 on mental health	29	7.1	0	0	0	0	0	7.1	0
	Not being able to visit patients in hospital, especially those about to die, and its impact on mental health	2	0.5	0	0	0.5	0.5	0	0.5	0
	Impact of COVID-19 on social health of students in schools	8	2.0	0	0	0	2.0	0	2.0	0
	Impact of COVID-19 on social health because of relationships between teachers, students and families	1	0.2	0	0	0	0.2	0	0.2	0
	Impact of COVID-19 on social health of students outside school	16	3.9	0	0	0	3.9	0	3.9	0
	Loneliness during lockdowns, especially for older people	2	0.5	0	0	0	0.5	0	0.5	0
	COVID-19 and work-life balance: lockdowns have negative impact while teleworking has positive impact	2	0.5	0	0	0.5	0.5	0	0.5	0.5
Total category 2	**14**	**104**	**15.8**	**0**	**1.7**	**2.7**	**7.6**	**1.7**	**25.4**	**0.5**
3. Infrastructures and waste management	Ineffective implementation of social distancing measures due to limited spaces in schools	3	0.7	0	0.7	0.7	0.7	0	0	0
	Difficulties for effective implementation of ventilation measures: lack of flexibility with type of activity, combined with usage of warm clothes, capacity limitations, etc.	12	3.0	0	0	3.0	0	0	0	0
	Insufficient use of outdoor spaces for educational activities and for communicating with parents	6	1.5	1.5	1.5	0	0	0	1.5	1.5
	Insufficient use of sustainable options for transport, which have increased during the pandemic (e.g., bicycle use)	2	0.5	0	0.5	0	0	0	0.5	0.5
	Services for young people, such as libraries, closed	1	0.2	0.2	0.2	0	0.2	0	0.2	0
	Waste from COVID-19 prevention measures	3	0.7	0	0.7	0	0	0	0	0
Total category 3	**6**	**27**	**6.6**	**1.7**	**3.6**	**3.7**	**0.9**	**0.0**	**2.2**	**2.0**
4: Communication and education for prevention	Ineffective communication: confusing, too focused on risks, contributing to stigma of certain age groups, non-transparent, non-evidence-based, overloading, inaccessible to certain groups	32	7.9	7.9	0	0	7.9	0	0	7.9
	Vaccine hesitancy due to concerns about vaccine side effects and low effectiveness^b^	12	3.0	3.0	0	0	0	0	3.0	3.0
	Lack of literacy to fight against anti-vaccine movements^b^	12	3.0	3.0	0	0	0	0	0	3.0
	Lack of awareness of the pandemic’s consequences to realize the importance of complying with regulations	29	7.1	7.1	0	7.1	0	0	0	0
	Misinformation, denialism and lack of skills to distinguish between evidence-based and non-evidence-based information and for shared responsibility	17	4.2	4.2	0	0	0	0	0	4.2
	Lack of knowledge on how to maintain hygiene in spaces and materials such as digital technologies	5	1.2	1.2	1.2	0	0	0	0	0
	Online classes are least effective and cause more fatigue	31	7.6	7.6	0	0	7.6	0	0	7.6
	Need to strengthen interaction between parents and students around learning	1	0.2	0.2	0	0	0.2	0	0	0.2
	Lack of participation of students and parents in decisions about changes in the educational model	3	0.7	0.7	0	0.7	0.7	0	0	0.7
	Need to maintain the reduced number of students per class during the pandemic	4	1.0	1.0	0	1.0	1.0	0	0	0
	Complementary non-formal education activities cancelled	5	1.2	1.2	0	0	1.2	0	1.2	0
	Teachers overloaded with new prevention roles	1	0.2	0	0	0	0.2	0	0.2	0
Total category 4	**12**	**152**	**37.3**	**37.1**	**1.2**	**8.8**	**18.8**	**0**	**4.4**	**26.6**
5: Social inequalities	Impact of COVID-19 on social inequalities	35	8.6	8.6	0	0	0	8.6	8.6	8.6
	Inequalities in accessibility to information and prevention measures (masks, antigen tests, etc.)	5	1.2	1.2	0	0	0	1.2	1.2	1.2
	Impact of COVID-19 on local trade	9	2.2		0	0	0	2.2	0	0
	Impact of digital divide during COVID-19	29	7.1	7.1	7.1	0	0	0	0	0
Total category 5	**4**	**78**	**19.1**	**16.9**	**7.1**	**0.0**	**0.0**	**12**	**9.8**	**9,8**
Totals	**40**	**406**	**89.9**	**64.8**	**13.6**	**26.3**	**36.4**	**13.7**	**41.8**	**49.5**

^a^ This measure may not always be applicable, because the measures need to be adjusted to the epidemiological situation, which is not always equal between different education levels.

^b^ Items related to the anti-vaccine movement were counted twice because the problems identified in these clusters were relevant for both education and health categories.

The categories in which participants identified the most issues were “Communication and education” (152 items) and “Physical, mental and social health” (104 items). The other categories ranked from “Social inequalities” (78 items) to “Participation of the education community and other stakeholders” (45 items) to “Infrastructures and waste management” (27 items).

**FIGURE 2:**
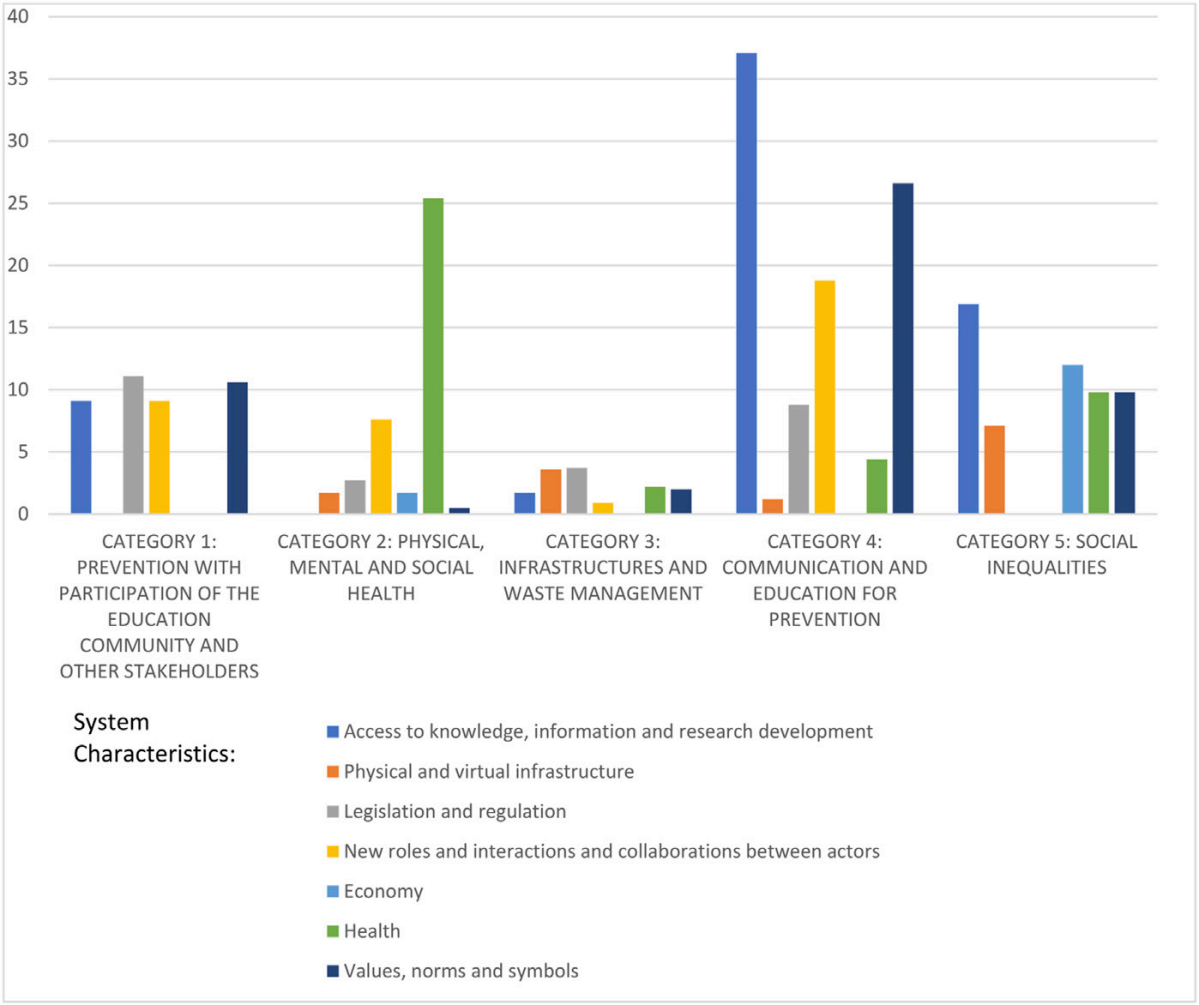
Weighted average by percentage of items in each system characteristic within each category. System-Oriented Dialogue Model, Catalonia, Spain, 2021.

The identified problems and needs covered the range of system characteristics. All categories had items in more than four characteristics, thus showing a relatively high complexity in every category (see Figure 2). Figure 2 also shows that the same system characteristics are key in several categories and they are therefore cross-cutting. For instance, in the category “Physical, mental and social health,” the cluster on the “Impact of COVID-19 on social health of students outside schools” has 16 items involving the system characteristic of “New roles and interactions and collaborations between actors.” This system characteristic is also important in the categories “Communication and education for prevention” and “Participation with the education community and other stakeholders.”

The key characteristic “Values, norms and symbols” is present in all categories, although it was most prominent in the following clusters of Categories 1, 4 and 5.

• “Lack of participation in decision-making at political level and within schools and other environments to adapt regulations to each context, to promote co-responsibility and to reach consensus on the needs for R&I,” with norms regarding decision-making capacity—the administration’s versus the citizens’ role in decision making—and fragmented versus collaborative governance models among different system characteristics (9.1% weight in Category 1).
• “Ineffective communication: confusing, too focused on risks, contributing to stigma of certain age groups, non-transparent, non-evidence-based, overloading, inaccessible to certain groups,” with values and norms regarding the lack of critical analysis of information or to stigmas against certain age groups for not following the rules (7.9% weight in Category 4). “Online classes are less effective and cause more fatigue,” with norms regarding the education model being teacher-centred versus cooperative learning (7.6% weight in the same category).
• “Impact of COVID-19 on social inequalities,” with norms regarding fairness and social justice (8.6% weight in Category 5).

### Recommendations

The first thematic analysis resulted in 47 recommendations. During the second thematic analysis, this was reduced to 8 thematic and 6 process-oriented final clusters of recommendations for the accompanying measures schools are supposed to address for more integrated prevention interventions (see Table 3).

**TABLE 3:** Categories and final recommendation clusters for accompanying measures, including thematic and process-oriented clusters. System-Oriented Dialogue Model, Catalonia, Spain, 2021.

Categories	Final thematic recommendation clusters	Final process-oriented recommendation clusters
Participation of the education community and other stakeholders (i.e., teachers, students, parents, healthcare providers, administration, community partnerships, etc.)		a. **Integrated policy support** aligned with different administrative departments and school needs (health, education, social and digital inequalities, infrastructures, R&I) at different geographical levels
		b. **Networks** with decentralized and collaborative organizational models (within and among schools and local communities)
		c. **Stakeholder participation in sharing responsibility**, helping administrations and schools adapt measures and R&I to their needs and geographical zones
		d. **Integrated and decentralized monitoring** within schools and families (health, compliance with measures, education, social and digital inequalities, infrastructures, R&I)
Physical, mental and social health	1. **Adapting norms** to better respond to problems and needs in implementing non-pharmaceutical measures	N/A
	• Ventilation protocols	
	• Social distancing in schools with limited spaces, flexibility for cohort groups	
	• Social distancing outside schools (between families and friends, in hospitals and residences, in transport, in services and non-formal education activities to avoid their closure, etc.)	
	• Masks (zero use and commercialization of non-certified masks, use of masks that facilitate non-verbal communication and breathing and that do not hurt ears, etc.)	
	• Hygiene protocols (cleaning spaces, disinfecting materials to avoid banning their sharing, washing hands, etc.)	
	2. **Pharmaceutical measures**: fast, easy and equitable access to prevention and diagnostic tools, improve effectiveness and reduce side effects	
	3. **Health literacy and promotion** challenges: knowledge, skills (i.e., distinguishing between evidence-based and non-evidence-based information) and attitudes (e.g., shared responsibility), including specific interventions on	
	• Mental health (access to psychological support, resources and techniques, early diagnosis)	
	• Social health (decentralized organization of social activities, responsible use of communication and information technologies (CIT), communication affected by masks, better work-life balance)	
	• Physical health (compliance with non-pharmaceutical measures and responsible use of pharmaceutical measures such as vaccination and diagnostic tools, physical exercise, healthy and sustainable diets, and skin and vocal cords care due to effects of masks)	
Infrastructures and waste management	4. **Improve infrastructures and use outdoor spaces** to comply with social distancing measures	N/A
	5. Reduce waste	
Communication and education for prevention	6. **Risk communication**: direct communication with experts, diversity of channels, official trustable channel, regulations to avoid *fake news*, involve students’ influencers, transparent messages that balance between risks and benefits of complying with measures and vaccinations and that do not stigmatize social groups, etc.	e. **Education**: education models with cooperative engagement of schools and families, balanced between online and face-to-face teaching, reduced teacher-student ratios per class, reduced transport of books, etc.
Social and digital inequalities	7. Fast, easy and equitable **access to social services and CITs**	f. Mutual support
	8. Local responsible consumption	

## Discussion

This study developed a System-Oriented Dialogue Model for designing future community partnerships that will facilitate participation while taking into account the systemic barriers for COVID-19 prevention in schools. The model, which is based on a structured participatory action-oriented process, includes a systems analysis to a) identify the key problems and (systemic) barriers that should be addressed and b) formulate recommendations for accompanying measures in order to achieve more integrated COVID-19 prevention that deals with the different system characteristics.

The problems and needs identified within each system characteristic illustrate the complexity of COVID-19 prevention. We classified the problems into five categories. Most issues fell into the “Communication and education” category, followed by the “Physical, mental and social health” and “Social inequalities” categories. Each category has a high degree of complexity, as evidenced by their having items related to at least four system characteristics. This complexity may explain why the categories of problems identified in our study have been persistent over a long period of time (13). These multifaceted and enduring—hence “wicked”—problems will require integrated interventions with coordinated approaches within and among all the categories. Single or multiple one-off interventions aimed at addressing problems in only one or some of these categories will simply not be effective enough (12).

Integrated interventions should consider the recommendations for each system barrier, including both the easily observable factors, such as “the lack of adapting NPI to each school reality,” and the less obvious ones, such as “values, norms and symbols” (e.g., norms regarding the education community’s role in the administration’s decision making processes). According to David Peter Stroh (14), the less obvious elements are key systemic factors that influence the more tangible elements.

Our study’s results also give insight into how schools can implement other international policy standards, such as those suggested by the WHO and UNESCO in “Global Standards and Indicators for Health Promoting Schools” (HPS) (3, 4).

Our System-Oriented Dialogue Model—a combination of the Dialogue Model and a systems analysis method—has been shown to be an effective methodology for including and integrating the experiential knowledge of teachers, students and their parents and for dealing with the complexity within the different problems and needs. To move towards the subsequent phases of our model that were not included in this study (i.e., prioritization, programming and implementation), we suggest that the community partnership should engage an even wider diversity of stakeholders at local and regional levels, including STEAM professionals in private and public organizations, policymakers, civil society organizations and the wider community with expertise in COVID-19 prevention and in the five problem categories. Stakeholders should be organized into different innovation or mission teams, each based on one of the final thematic recommendation clusters and all follow the final process-oriented recommendation clusters. The management structure should be defined with participatory methodologies. For the more complex clusters, such as the promotion of mental health, physical exercise or healthy and sustainable diets, stakeholders should conduct a new iteration of the System-Oriented Dialogue Model to deepen the system analysis and to validate the recommendations through broader collective transformation knowledge. The final output of this new iteration could be Research, Innovation and Action (RIA) plans with collaborative and decentralized approaches for implementing the most complex recommendations. Finally, the innovation teams could implement the resulting RIA plans with all stakeholders participating. However, further research is needed to explore how to improve: a) the system analysis to better consider the interactions between the different items and stakeholders, b) the involvement of the students and their parents in the different phases of the research process and c) the motivation of the participants for instances by introducing a theory of change. Further research involving more schools, both in our context and beyond, is also needed to validate our results, as some specificities may arise.

### Policy Implications

Our results show the urgent need to establish community partnerships, as suggested by the ECDC, UNESCO and WHO, to adapt and implement integrated interventions that act simultaneously in the different categories.

However, implementing engagement processes such as our System-Oriented Dialogue Model is not straightforward. We therefore recommend that schools are provided clear guidelines for all the phases of our model, including those not addressed in this study. These should include participatory ideation processes that use system innovation methodologies for the programming and implementation phases (11, 12, 14, 15). The guidelines should be supported with training packages to enable schools to engage their students and key stakeholders in implementing recommendations as integrated interventions using collaborative and decentralized approaches. To ensure that schools dedicate efforts to implementing these guidelines, they must be designed with participatory approaches that not only improve health protection and promotion but also the learning of science (16). The Open Schooling movement has demonstrated that one key to improving science learning is promoting learning activities in which students collaborate with scientists, families and other stakeholders to contribute to solving social challenges through research. The guidelines should therefore be designed following these principles, and they should be adjustable and adaptable to local needs. This means they should be based on participatory research approaches. Besides contributing to learning and implementation, such approaches would allow the education community to participate in the necessary research to deal with the complexities within each category. The interaction between schools and scientists would also help the scientific community to better adapt their research to the needs and expectations of schools, which is a key policy priority of many governmental organizations willing to increase the impact of research (16–19).

The scientific community could act as a facilitator by a) supporting the use of system-oriented participatory research approaches and b) using such approaches to monitor the process, the knowledge generated and the learning and changes within each category. This would enable reflexivity and help participants deal with uncertainties and conflicts while challenging current practices and related institutions (20). At the same time, it would allow the integration and management of research data at different levels: class, school, local, regional.

Implementations should be supported by national and regional policies or strategies “that recognise Health Promoting Schools as key vehicles to achieve national development goals through education and provide a framework for nation-wide promotion of these schools” (3, 4). They should also be coordinated with key international policies in other fields, such as those of the ECDC (2), Open Schooling (21, 22) and organizations willing to increase the impact of research (18). This requires “dedicated resources for true partnerships” (2).

### Conclusion

The current model of health protection and promotion in schools needs to use more systemic and collaborative approaches that could be designed with the System-Oriented Dialogue Model described here. This model been useful in designing strategies that community partnerships can use to implement NPI for COVID-19 prevention, including the ECDC’s “accompanying measures.” As we discussed, our results are aligned with international policy standards for Health Promoting Schools and Open Schooling. However, for effective and sustainable implementation, coordinated policy support is required at different geographical levels with dedicated resources to achieve evidence-based impacts on both health promotion and education.

## Group Members of the Sentinel School Network Study Group of Catalonia

Principal investigators: Jordi Casabona [Centre d’Estudis Epidemiològics sobre les Infeccions de Transmissió Sexual i Sida de Catalunya (CEEISCAT)-CIBERESP], Josep Basora (Institut Universitari d'Investigació en Atenció Primària (IDIAP Jordi Gol). Project manager: Anna Bordas (CEEISCAT). Technical committee: Jordi Casabona (CEEISCAT), Jordi Sunyer (ISGlobal), Antoni Soriano (Hospital Universitari Vall d’Hebron), Rosina Malagrida (Living lab for Health, IRSICaixa) as Work Package coordinators. Cinta Folch, Pol Romano, Cristina Sánchez, Esteve Muntada, Anna Bordas, Andreu Colom-Cadena i Jordi Casabona (CEEISCAT), Mireia Gascón, Maria Subirana, Jordi Sunyer (ISGlobal), Rosina Malagrida, Jessica Fernández (Living lab for Health at IrsiCaixa), Antoni Soriano (Hospital Universitari Vall d’Hebron). Microbiology laboratories: Tomàs Pumarola, Andrés Antón, Cristina Andrés, Juliana Esperalba, Albert Blanco (Hospital Universitari Vall d’Hebron), Ignacio Blanco, Pere-Joan Cardona, Maria Victoria González, Gema Fernández, Cristina Esteban (Hospital Universitari Germans Trias i Pujol) Data Management and statistical analysis: Yesika Díaz, Lucia Alonso, Jordi Aceiton, Marcos Montoro (CEEISCAT). Data Protection Officer and Technical Support: Esteve Muntada (CEEISCAT). Communication manager: Pol Romano, Cristina Sánchez (CEEISCAT). Field team: Maria Subirana (ISGlobal), Jessica Fernández (Living Lab for Health, IRSICaixa), Andreu Colom-Cadena, Isabel Martínez, Marina Herrero, Alba García, Juan Rus, Paula Ribas, Alba Blanco (CEEISCAT). Community Paediatricians: Esperança Macià i Silvia Burgaya (CAP Manlleu), M^a^ Teresa Riera-Bosch, Elisabet Sola (EAP Vic Nord), Lidia Aulet, Maria Mendoza, Lidia Busquets (EAP Vic Sud), Xavier Perramon, Júlia Sebastià (EAP Eixample Dret), Ana Moreno (Cap Ripollet), Xavier Duran, Belen Pérez (EAP Can Gibert del Pla), Anna Gatell (Equip Territorial de Pediatria Alt Penedès), Maria Coma (Hospital Universitari Joan XXIII). Epidemiology suveillance services: Marc Olivella, Irene Barrabeig, Ignacio Parro’n, Mònica Carol, Núria Bes, Pere Godoy, Sofia Minguell, Maria Rosa Sala.
