# Supplementary material for: A System-Oriented Dialogue Model to Design Community Partnerships for More Effective Sars-Cov-2 Prevention in Schools: The Case of Spain
Source: Int J Public Health. 2023 May 2;68:1605624. doi: 10.3389/ijph.2023.1605624 (PMC10186344; doi:10.3389/ijph.2023.1605624)
Supplement: Supplementary file 1 [file Table1.pdf]

International Journal of Public Health

Article title: A System-Oriented Dialogue Model to initiate community structures for more effective Sars-Cov-2 prevention in schools: The case of Spain

Supplementary File 1. Table with clusters of problems, recommendations and clusters of recommendation to improve SARS-Cov-2 prevention in schools. The recommendations are targeted at different stakeholders (students, teachers, cleaning staff, administrative staff, families, health care professionals, citizens, etc.). System-Oriented Dialogue Model, Catalonia, Spain, 2021.

Clusters of problems	Recommendations		Examples of solutions:	Clusters of recommendations
**1. PARTICIPATION OF THE EDUCATION COMMUNITY AND OTHER STAKEHOLDERS**				
**Lack of participation in decision-making at political level and within schools and other environments to adapt regulations to each context, to promote co-responsibility and to reach consensus on the needs for R&I**<br><br>Students need to transport a lot of books due to COVID-19 measures	#1	Facilitate participatory spaces for adapting prevention measures to the needs of the education system	Examples of prevention measures to be adapted with participation:<br>• Hospital protocols: allow minors to accompany adults, for example to visit COVID-19 patients in critical condition<br>• Confinement regulations: ensure the elderly are not left unattended<br>• Use of facemasks[1]<br>• Balance between face-to-face and virtual education<br>• Avoid book transport for teleworking/online education, for example by promoting using digital books with a good balance between paper and digital education<br>• Ventilation protocols<br>• Distancing measures in schools, considering infrastructure realities, giving more flexibility to cohort	**Stakeholder participation** in administration and schools to adapt measures to needs and geographical zones, **sharing responsibility** in education, social and digital inequalities, infrastructures, R&I** and health** (non-pharmaceutical measures: ventilation, social distancing in schools with limited spaces, cohort groups, social distancing outside schools (between families and friends, in hospitals and

[1] Scientific community contribution: If we show, as already suggested by evidence, that transmissibility is very low (only 1% inside schools) and that students who are index cases rarely transmit the infection to the cohort group, this recommendation could be re-evaluated in the future.

Norms are not adapted to the COVID-19 prevalence in different geographic zones<br><br>Norms are not equitable between education levels*<br><br>Not being able to visit patients in hospital, especially those about to die, and its impact on mental health			groups, mobility and curfews, and allowing sharing of materials prior to disinfection	residences, etc.), balance between online and face-to-face teaching, transport of books, etc.)<br><br>**Integral policy support** aligned among different administration departments and school needs (health, education, social and digital inequalities, infrastructures, R&I) at different geographical levels
	#2	Promote policies that help increase investment in research for vaccines and medicines, including research on side effects, to fight the anti-vaccination movement	• Promote R&I to improve the effectiveness of vaccines and minimize possible adverse effects	
	#3	Promote co-responsibility in R&I for COVID-19	• Promote citizen participation policies in R&I	
	#4	Promote adapting regulations to the indicators of prevalence of the pandemic in different geographical areas[2]		
	#5	Promote equitable regulations between educational levels[3]*		
	#6	Promote better coordination between different administration departments	• Coordinate prevention strategies based on criteria accepted by different departments at local, national and international levels	**Integral policy support** aligned among different administration departments and school needs (health, education, social and digital inequalities, infrastructures, R&I) at different geographical levels
COVID-19 and work-life balance (lockdowns have negative impact, while teleworking has positive	#7	Minimize the impact on work-life balance during confinements[4]	• Modify regulations for confinement of students who have been contacts and have tested negative with a PCR test<br>• Encourage companies to facilitate teleworking and work-life balance	Better **work-life balance**

[2] Scientific community contribution: Evidence has shown that the school reflects the incidence of COVID in the community, so this is a very applicable recommendation.

[3] Scientific community contribution: Regulations must respond to the epidemiological situation of each educational level; therefore, this recommendation would not always be applicable.

[4] Scientific community contribution: There have been several positive cases at the end of the confinement period. We find it difficult to end quarantine with a PCR test.

impact, more flexible confinements, etc.)				
Failure to comply with prevention measures	#8	Improve regulations to control measures compliance	• Assign specialized personnel such as specialized patrols	Integral and decentralized **monitoring** within schools and families (health, measures compliance, education, social and digital inequalities, infrastructures, R&I)
**2. PHYSICAL, MENTAL AND SOCIAL HEALTH**				
Impact of COVID-19 on:<br>• mental Health<br>• physical exercise<br>• screen addictions<br>• weight gain and obesity<br>• social health inside and outside schools<br><br>Loneliness during lockdowns, especially for older people<br><br>Impact of hydroalcoholic gels on the skin<br><br>Inconveniences of using masks (on communication, hygiene, breathing, pressure on ears	#20	Investigate the pandemic's impact on health in the education system[5]	• On screen addictions<br>• On emotional well-being<br>• On weight gain and obesity<br>• On other pathologies	Integral and decentralized **monitoring** within schools and families (impact of prevention on health, measure compliance, education, social and digital inequalities)
	#21	Improve the emotional well-being of teachers, students and families	• Offer (human) resources, programs, spaces and tools for training, support and monitoring of the state of the school and its interventions<br>• Facilitate access to health services<br>• Provide relaxation/meditation techniques	
	#22	Facilitate the organization of social interaction activities in schools, supported by the health sector	• Provide diagnostic tests to schools	Health literacy and **Health Promotion** interventions promoting knowledge, skills (i.e., distinguishing between evidence-based and non-evidence-based
	#23	Explore, in a participatory way with the education community, if it is necessary to regulate when to use masks	• Using masks with the following characteristics: transparent to facilitate non-verbal communication, mentholated to avoid bad odours, materials that facilitate breathing, elastic bands that do not harm ears, etc.	

[5] Scientific community contribution: This is an issue of great concern, and diverse studies have already been carried out. They showed that during the pandemic period, students decreased their physical activity during confinement and increased their screen time, spending too much time with video games and other electronic media.

and vocal cords, etc.) for the different stakeholders<br><br>Impact of COVID-19 on social health because of relationships between teachers, students and families				information), attitudes (e.g., shared responsibility) and specific interventions on:<br>• Mental health** (access to psychological support, resources and techniques, early diagnosis)<br>• Social health (decentralized organization of social activities**, responsible use of CIT*, communication problems due to masks)<br>• Physical health (benefits and risks of compliance with measures and vaccination, physical exercise**, healthy and sustainable diets**, skin and vocal cords care due to mask usage)<br><br>**Hygiene protocols**: cleaning spaces, disinfecting materials to
	#24	Improve emotional support for families with COVID-19	• Offer psychological support	
	#25	Promote healthy habits within both schools and families	• Provide guidelines with recommendations for physical activities and descriptions of benefits for different age groups	
	#26	Regulate the use of hydroalcoholic gel and promote the use of products to minimize their impact on skin	• Creams to avoid dry skin due to gels	
	#27	Provide solutions to avoid damaging vocal cords due to mask usage	• Provide microphones to teachers<br>• Market only approved masks	
	#28	Prevent screen addictions, especially among teenagers	• Effective campaigns and other awareness-raising measures<br>• Explore in a participatory way whether it is necessary to define regulations for responsible screen use	
	#29	Facilitate socialization outside schools	• Promote families supporting people in their social and family environment, especially the elderly and those that feel lonely<br>• Provide virtual environments	
	#30	Minimize boredom for people who are confined alone in isolation	• Provide digital platforms for free	

				avoid banning the sharing of materials, cleaning hands<br><br>Zero use and commercialization of **non-certified masks**
	#31	Facilitate socialization within schools so students can know and interact with other students from different cohorts, especially outdoors	• Work in cooperative groups – see innovation in the educational model<br><br>• See measures to adapt distancing rules within schools	New models of (online) **education** with cooperative learning within schools and families**<br><br>**Stakeholder participation** in administration and schools to adapt measures to needs and geographical zones, **sharing responsibility** in education, social and digital inequalities, infrastructures, R&I and health** (non-pharmaceutical measures: ventilation, social distancing in schools with limited spaces, cohort groups, social distancing outside schools, balance between online and face-to-face teaching, transport of books, protocols in hospitals, etc.)

It takes too long to receive PCR test results	#32	Promote policies that facilitate rapid vaccine production	• Easier and faster access to prevention and diagnosis tools	Fast, easy and equitable **access** to prevention, diagnostic tools and CIT tools**
	#33	Faster access to diagnostic test results		
**3. INFRASTRUCTURE AND WASTE MANAGEMENT**				
Ineffective implementation of social distancing measures due to limited spaces in schools	#34	Adapt spaces to allow better compliance with social distancing measures	• Improve school infrastructures	Improve **infrastructures** to comply with social distancing measures
**Difficulties for effective implementation of ventilation measures: lack of flexibility with sort of activity, combined with usage of warm clothes, capacity limitations, etc.**	#35	Improve ventilation measures in classrooms even when the pandemic is over	• Install permanent CO_2 detectors[6]**<br>• Promote the maintenance of good ventilation habits that are flexible in different circumstances such as: exams when there is playground noise, when it is cold outside (i.e., with recommendations to increase the use of warm clothing), etc.<br>• Define capacity limitations for each room to reduce the risk of COVID-19 and other infections	Improve **ventilation** habits and use of outdoor spaces
Insufficient use outdoor spaces for educational activities and for communicating with parents	#36	Promote the rotation of spaces and the use of outdoor learning spaces		Improve **ventilation** habits and use of outdoor spaces
	#37	Propose alternative school-family interaction mechanisms to facilitate communication	• Outdoor walks<br>• Conducting interviews with families on the playground	
Insufficient use of sustainable options for transport, which have increased during the pandemic (e.g. bicycle use)	#38	Promote sustainable transport that also facilitates compliance with prevention and security measures	• Collaborative citizen initiative to accompany groups of students by bicycle	More sustainable and collaborative **transport**
Services for young people, such as libraries, closed	#39	Ensure that libraries and other public and private infrastructures and activities aimed at children	• Adapting services to comply with prevention measures	**Services** and non-formal education kept open

[6] Scientific community contribution: Installing permanent sensors would likely be too expensive to be cost-effective. Efforts should therefore go towards raising awareness (with temporary use of sensors) on the need for promoting cross ventilation whenever possible.

		and young people are kept open		
Waste from COVID-19 prevention measures	#40	Promote measures to reduce, reuse or recycle waste from prevention measures, such paper used for disinfection, masks, etc.	• Carry out awareness campaigns that include, for example, talks and support by teachers<br>• Provide specific collection containers	**Reduce waste** from prevention measures
**4. COMMUNICATION AND EDUCATION FOR PREVENTION**				
**Ineffective communication: confusing, too focused on risks, contributing to stigma of certain age groups, non-transparent, often non-evidence-based, overloading, inaccessible to certain groups**<br><br>**Lack of literacy to fight against anti-vaccine movements****<br><br>**Concerns about vaccine side effects and low effectiveness****<br><br>**Lack of awareness of the pandemics' consequences to realize the importance of complying with regulations**	#9	Improve the communication of information so it is clear, concise, objective, understandable, and evidence-based	• Promote direct communication between experts and teachers<br>• Use diversity of channels<br>• Involve opinion leaders or *leading influencers* of young people, with prior consensus from the educational community<br>• Disseminate messages focused on:<br>○ safety to better counterbalance those on risk and mortality<br>○ avoiding stigmatizing certain groups, such as young people, who have not been the only ones to not always strictly follow prevention measures<br>○ benefits of regulations (i.e., use of masks)<br>○ transparent information regarding the risks and benefits of vaccines that contributes to overcoming fear of vaccines and fighting the anti-vaccination movement<br>• Regulate the issuance of confusing and unverified information, such as *fake news*[7], with a certificate of verified information and the obligation to cite sources	Risk **Communication** (direct communication with experts, diversity of channels, official trustable channel, influencers for students, transparent messages that balance between risks and benefits of compliance with measures and vaccinations but without stigmatizing social groups, regulations to avoid *fake news*)<br><br>Health literacy and **Health Promotion** interventions promoting knowledge, skills (i.e., distinguishing between evidence-based and non-evidence-based information), attitudes (e.g., shared responsibility) and

[7] Scientific community contribution: COVID-19 has led to the generation of a lot of information, and, unfortunately, we must live with *fake news*. However, we need to empower the education community with skills to discern between evidence-based and non-evidence-based information.

			• Create an official channel of communication (i.e., website) dedicated exclusively to informing citizens on pandemic-related aspects	specific interventions on:<br>• Mental health** (access to psychological support, resources and techniques, early diagnosis)<br>• Social health (decentralized organization of social activities**, responsible use of CIT**, communication problems due to masks)<br>• Physical health (benefits and risks of compliance with measures and vaccination, physical exercise**, healthy and sustainable diets**, skin and vocal cords care due to mask usage)
**Online classes are least effective and cause more fatigue**	#10	Promote innovation in pedagogical approaches that contribute to improved learning without losing the current effective methodologies	• Collaborative learning methodologies	New models of (online) **education with cooperative learning** within schools and families

	#11	Regulate the online teleworking/education of teachers and students	• Guide with recommendations such as: limiting screen time, behaviour guidelines, reducing group size, promoting active learning, configuring screen lighting to minimize fatigue, etc.	**Guidelines** for online education and teleworking
Need to strengthen interaction between parents and students around learning	#12	Promote families supporting students' learning process	• Promote educational activities that encourage interaction with families	
**Misinformation, denialism and lack of skills to distinguish between evidence-based and non evidence-based information and for shared responsibility**	#13	Fight against misinformation, denialism, conspiracy theories and opinions not based on evidence	• Promote educational activities that contribute to the development of skills that help to distinguish between evidence-based and not evidence-based information	Health literacy and **Health Promotion** interventions promoting knowledge, skills (i.e., distinguishing between evidence-based and not evidence-based information), attitudes (e.g., shared responsibility) and specific interventions on:<br>• Mental health** (access to psychological support, resources and techniques, early diagnosis)<br>• Social health (decentralized organization of social activities**, responsible use of CIT**, communication
	#14	Promote that the rules are respected because of responsibility and not obligation both inside and outside the school environment	• Promote awareness and teamwork	
Lack of knowledge on how to maintain hygiene in spaces and with materials such as digital technologies	#15	Provide recommendations on how to clean spaces and materials such as digital devices.	• Cleaning protocols	

				problems due to masks)<br>• Physical health (benefits and risks of compliance with measures and vaccination, physical exercise**, healthy and sustainable diets**, skin and vocal cords care due to mask usage)<br><br>**Hygiene protocols**: cleaning spaces, disinfecting materials to avoid banning their sharing, cleaning hands
Lack of participation of students and parents in decisions about changes in the educational model	#16	Promote students and families evaluating schools, including the measures adopted during the pandemic		Integral and decentralized **monitoring** within schools and families (health, measure compliance, education, social and digital inequalities, infrastructures, R&I)
Need to maintain the reduced number of students per class during the pandemic	#17	Promote and maintain the reduced numbers of students per class when the pandemic ends, at least for the most difficult subjects	• Support for splitting groups<br>• Fewer students in the physical and virtual classrooms	Reduced **numbers** of students per class
Complementary non formal education activities cancelled	#18	Promote adapting complementary activities to respect the	• Tours, visits, trips	**Services** and non-formal education kept open

		restrictions and avoid cancellations	• Laboratory workshops<br>• Out-of-school activities	
Teachers overloaded with new prevention roles	#19	Involve security and administration personnel in the supervision and application of prevention measures		**Networks** with decentralized and collaborative organizational models (within and among schools and local communities)*
**5. SOCIAL INEQUALITIES**				
**Impact of digital divide during COVID-19**	#41	Promote policies to combat the digital divide for schools, teachers, students and families	• Facilitate computer devices and ensure connectivity<br>• Provide training (personalized, multilingual and with innovative formats such as promoting peer learning, including programming to develop educational tools)	**Social inequalities** and digital divide**<br><br>Fast, easy and equitable **access** to prevention, diagnostic tools, CIT tools and social services**<br><br>**Networks** with decentralized and collaborative organizational models (within and among schools and local communities) **
**Impact of COVID-19 on social inequalities**	#42	Facilitate equitable access to prevention measures	• Provide masks and diagnostic tests such as PCRs at reasonable prices or even for free	
	#43	Ensure the reduction of social inequalities that have become more visible during the pandemic, facilitating a process of systemic change	• Aid for families who require it<br>• Minimizing school segregation by promoting the equitable distribution of students in vulnerable situations between schools<br>• Other actions to ensure systemic change	
	#44	Facilitate access to effective social services during pandemics	• To labour insertion services<br>• To economic aid (especially for affected sectors)	
Inequalities in accessibility to information and prevention measures: masks, antigen tests, etc.	#45	Promote schools to collaborate with local entities to inform families about the most vulnerable environments	• Collaboration with non-formal education, leisure and social entities, etc.	
Impact of COVID-19 on local trade	#46	Promote the autonomy of education centres for the budget management in the benefit of local commerce	• Protocols for responsible local consumption<br>• Purchase of computer products by each school<br>• Prioritize the use of free software	Responsible local **consumption**

	#47	Explore in a participatory way if it is necessary to limit the consumption of scarce products in pandemic situations		

*Items not considered in the clustering, because they were rejected by the scientific community.

**Complex recommendations that will require a new iteration of the System-Oriented Dialogue Model.
